# Polyphenols as Possible Agents for Pancreatic Diseases

**DOI:** 10.3390/antiox9060547

**Published:** 2020-06-23

**Authors:** Uroš Gašić, Ivanka Ćirić, Tomislav Pejčić, Dejan Radenković, Vladimir Djordjević, Siniša Radulović, Živoslav Tešić

**Affiliations:** 1 Department of Plant Physiology, Institute for Biological Research “Siniša Stanković”, National Institute of Republic of Serbia, University of Belgrade, Bulevar Despota Stefana 142, 11060 Belgrade, Serbia; uros.gasic@ibiss.bg.ac.rs; 2 Innovation Center, University of Belgrade—Faculty of Chemistry, P.O. Box 51, 11158 Belgrade, Serbia; ivankai@chem.bg.ac.rs; 3 Clinic of Urology, Clinical Centre of Serbia, Pasterova 2, 11000 Belgrade, Serbia; tomislav.pejcic@gmail.com; 4 University of Belgrade—Faculty of Medicine, dr Subotića 8, 11000 Belgrade, Serbia; dejanr09@yahoo.com; 5 First Surgical Clinic, Clinical Center of Serbia, Koste Todorovića 6, 11000 Belgrade, Serbia; vladimir.djordjevic@kcs.ac.rs; 6 Institute for Oncology and Radiology of Serbia, Pasterova 14, 11000 Belgrade, Serbia; sinisar@ncrc.ac.rs; 7 University of Belgrade—Faculty of Chemistry, Studentski trg 12–16, P.O. Box 51, 11158 Belgrade, Serbia

**Keywords:** pancreatic diseases, polyphenols, synergistic effects, polyphenol bioavailability, quercetin, resveratrol

## Abstract

Pancreatic cancer (PC) is very aggressive and it is estimated that it kills nearly 50% of patients within the first six months. The lack of symptoms specific to this disease prevents early diagnosis and treatment. Today, gemcitabine alone or in combination with other cytostatic agents such as cisplatin (Cis), 5-fluorouracil (5-FU), irinotecan, capecitabine, or oxaliplatin (Oxa) is used in conventional therapy. Outgoing literature provides data on the use of polyphenols, biologically active compounds, in the treatment of pancreatic cancer and the prevention of acute pancreatitis. Therefore, the first part of this review gives a brief overview of the state of pancreatic disease as well as the procedures for its treatment. The second part provides a detailed overview of the research regarding the anticancer effects of both pure polyphenols and their plant extracts. The results regarding the antiproliferative, antimetastatic, as well as inhibitory effects of polyphenols against PC cell lines as well as the prevention of acute pancreatitis are presented in detail. Finally, particular emphasis is given to the polyphenolic profiles of apples, berries, cherries, sour cherries, and grapes, given the fact that these fruits are rich in polyphenols and anthocyanins. Polyphenolic profiles, the content of individual polyphenols, and their relationships are discussed. Based on this, significant data can be obtained regarding the amount of fruit that should be consumed daily to achieve a therapeutic effect.

## 1. Pancreatic Cancer

### 1.1. General Trends

Pancreatic cancer has been perceived as an incurable disease worldwide. It is estimated that in 2020, pancreatic carcinoma (PC) will be fourth leading cause of death due to carcinoma in the world [1]. American Cancer Society states a 9% five-year relative survival rate for all stages combined, while for the patients diagnosed at a distant stage (accounting for 53%) five-year survival is 3% [2]. Despite the somewhat increase in PC five-year survival rate, the prognosis of patients with this type of cancer remains very poor. The main reasons for this poor prognosis are the fact that the disease is advanced at the presentation, the high risk of developing distant metastasis (being generally resistant to most chemotherapeutic agents), and the absence of sufficiently effective treatment strategies. Once diagnosed, surgery is the best choice for the therapy as is the case with the majority of solid tumors, while chemotherapy and radiotherapy are suitable for treatment of patients with locally unrespectable and recurrent PC with the purpose of improving the patient’s quality of life and gaining a modest survival benefit [3,4].

The relatively low percentage of curative resections of PC can be attributed to the late diagnosis, which occurs in most cases when cancer is in its advanced stage. However, even in the 10% of people diagnosed with local disease, five-year survival is less than 40% [2]. Genome sequencing has recently provided a time spam of pancreatic carcinogenesis. Namely, there is evidence of the existence of 12 signaling pathways that are not promoting any distant metastasis 20 years after the initial mutation [5,6]. These findings point to the importance of PC early detection for better disease prognosis. Hence, earlier tumor detection, histologically curative resection, and improvement of both preventive and treatment strategies are essential for long-term survival and healing of PC patients.

It is essential to further elucidate the precise biological and molecular mechanisms underlying pancreatic cancer development, identify the risk factors responsible for this disease, as well as to verify newer, more reliable prognostic factors that could predict the survival rate of these patients.

Exocrine tissue of the pancreas is the place of the development of majority PC (93%) while the remaining 7% are endocrine tumors known as pancreatic neuroendocrine tumors (NETs). The most common type of PC is adenocarcinoma while other types of exocrine carcinoma such as acinar cell carcinoma, an intraductal papillary mucinous neoplasm (IPMN), mucinous cystic neoplasm, etc., are much rarer [7].

### 1.2. PC Development

Cancer progression is genetically the consequence of the combination of oncogene activation and tumor suppressor inactivation. Likewise, it was established that pancreatic ductal adenocarcinoma (PDAC) development requires certain mutations; namely, mutation of the GTPase Kras (KRAS) oncogene and mutations of tumor suppressor genes, cyclidin-dependent kinase inhibitor 2A (CDK N2A), tumor protein p53 (TP53), a small mothers against decapentaplegic (SMAD) protein family member 4 (SMAD4) [8,9]. Additionally, increasing data have been pointing to epigenetic dysregulation, such as DNA methylation, histone acetylation, or microRNA changed expressions of tumor-associated genes such as silencing of the tumor suppressor p16 (ink4a), as genetic hallmarks of PC [10]. The majority of PDAC and early pancreatic intraepithelial neoplasias (PanIN) lesions that prograde into PDAC have mutated KRAS with the incidence of 70%–90% [11]. Substantial and rapidly accumulating evidence validates a key role of the following major effectors of KRAS in pancreas adenocarcinomas: serine/threonine-protein kinase (Raf) [12], phosphatidylinositol-4,5-bisphosphate 3-kinase (PI3K) [13], and Ral guanine nucleotide dissociation stimulator (RaLGDS) [14].

The most promising preclinical models of PDAC include KC mice, which express oncogenic KRAS from the earliest stage of pancreas development [15] and KPC mice, which represent KC mice crossed with mice with non-functional or mutant alleles of p53 gene [16]. The use of such models revealed that when subjected to inflammatory insult, PanIN with oncogenic KRAS could rapidly progress into PDAC [17], which can explain the fact that chronic inflammation is a well-established risk factor of PC [18]. Interestingly however, direct targeting of KRAS by inhibition has not given any results so far [11].

The molecular pathway that activates or counteracts oxidative stress has been also implicated in PC development [19]. Oxidative damage of lipids can lead to transformation of proteins, lipids, and DNA into toxic and mutagenic metabolites that alter cell homeostasis. Although genetic changes are responsible for the transformation into neoplastic phenotypes, reactive oxygen species (ROS) production certainly has its role in cancer development and contributes to changes in cancer cells that provide them with invasive and aggressive characteristics. Indeed, during the development and progression of PDA, oncogenic KRAS causes metabolic changes that lead to increased generation of ROS and upregulates antioxidant systems to balance ROS to levels at which they contribute to oncogenic transformation and tumor progression [20].

The role of exosomes in pancreatic cancer development has been gaining more and more attention. These extracellular vesicles, generated by the endocytosis and released by exocytosis, are the important mediators of intracellular communication [21]. Exosomes deliver carcinogenic proteins, specific miRNAs, cytokines, and adhesion molecules to cancer cells promoting cancer development [22]. Research has shown that exosome-mediated delivery of certain compounds such as miRNA can enhance proliferation, migration, and the specific gene expression of PC cells [23]. A recent review proposed potential functions of exosomes as novel diagnostic biomarkers and even their clinical application in PC treatment [24].

### 1.3. Risk Factors for PC Development

The identification of the high-risk population and appropriate measures for patients at risk is yet another strategy for PC prevention. Risk factors include multiple genetic syndromes and modifiable risk factors, which are of special importance since they can greatly increase the risk of PC development [25]. Chronic pancreatitis causes a 4% increase in cumulative risk of PC development [26], diabetes [27], and some infectious diseases (*Helicobacter pylori*, Hepatitis B virus, or Human Immunodeficiency virus) have also been listed as risk factors for PC development [28]. The main nongenetic (modifiable) environmental risk factors are exposures to various environmental pollutants such as cigarette smoke, nitrosamines, chlorinated hydrocarbon solvents, and toxic metals, i.e., arsenic and cadmium [29,30,31]. Further studies on modifiable risk factors, as well as the introduction and following of well-defined preventive measures, will provide novel insight for the earlier diagnosis and more effective surgical and therapeutic treatment of this disease.

Today, the very interesting treatment of PC is the potential use of polyphenols. Polyphenols have shown promising antitumor properties in numerous experiments on human PC cell lines and animal studies. In this article, we discuss the experimental and clinical data on polyphenols mainly from fruits as possible candidates for the prevention of cancerogenesis of PC. The use of various extracts and supplements might increase, at least, the quality of life of patients with metastatic PC.

## 2. Polyphenols

Polyphenols have been known for a long time to nutritionists and the scientific community, considering them the most powerful natural antioxidants. Namely, the cells of our body are often attacked by free radicals, and polyphenols are molecules that prevent the formation of free radicals, and are therefore the guardians of our overall health [32]. Since ancient times, humans consume large amounts of polyphenols within plant foods. Polyphenols are secondary plant metabolites, comprising over 4000 types of molecules, grouped into nine groups: flavonoids, isoflavonoids, aurones, chalconoids, flavonolignans, lignans, stilbenoids, curcuminoids, and tannins. Flavonoids can be further divided into six subclasses: flavones, isoflavones, flavonols, flavanones, flavanols, and anthocyanins [33].

**Figure 1:**
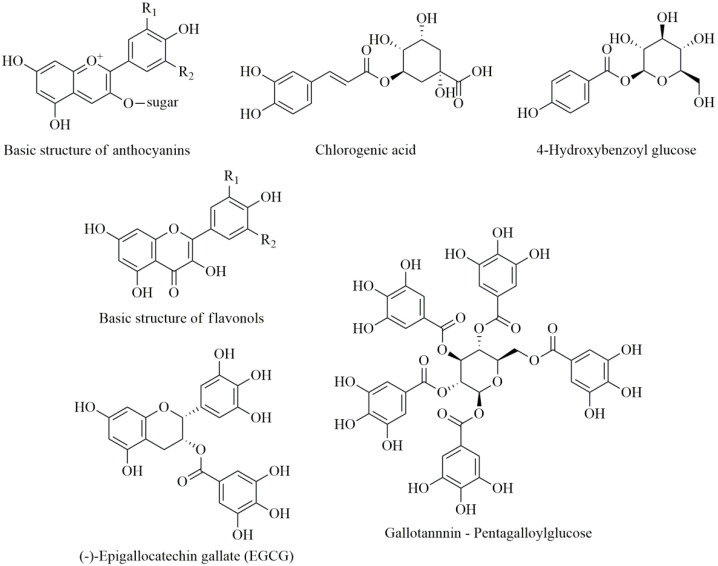
Chemical structures of some polyphenols commonly occurring in fruits.

There are certain fruits very rich in polyphenols, which are characterized by the intensity of their aroma, taste, and color [34]. For example, grapes, apples, blueberries, strawberries, blackberries, raspberries, aronia, and plums are the richest in polyphenols [33]. The most commonly occurring polyphenols in fruit are anthocyanins (in colored fruit), then hydroxycinnamic and hydroxybenzoic acids along with their derivatives, followed by catechins, tannins, and flavonols [35] (Figure 1).

**Figure 2:**
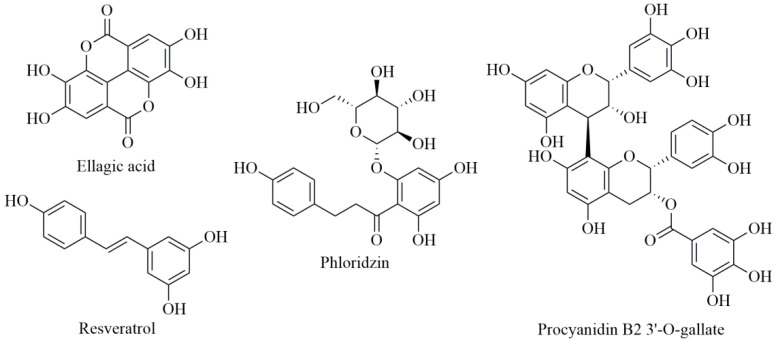
Chemical structures of ellagic acid, phloridzin, resveratrol, and procyanidin B2 3′-*O*-gallate.

Ellagic acid and its derivatives (elagitannins) are known to be present in various berries, especially in berry seeds [36,37]. Chalcones are polyphenolic compounds that are specific to apple fruits, while resveratrol and some of its stilbene derivatives are characteristic of grapes (especially found in red grape skins) [35], while the major polyphenols in grape seeds are proanthocyanidins [38] (Figure 2).

One of important dietary sources of hydroxybenzoic and hydroxycinnamic acids and dihydrochalcones are apples (*Malus domestica*). In harvested apple cultivars from China [39] the dominating phenolic acids were chlorogenic and protocatechuic acids, while in apple cultivars from California, the predominant phenolic acids were found to be chlorogenic and caffeic acids [40]. Investigation of apple pulp and peel [40] showed that apple pulp contains more phenolic acids than apple peel based on dry weight. Total chlorogenic acid content (based on dry weight) in apple pulp was in the range 150–740 mg/kg, and in apple peel, 63–450 mg/kg. Caffeic acid concentration (based on dry weight, DW) varied from 44–287 mg/kg in pulp and 1.4–125 mg/kg in apple peel [40]. Apple skin is suggested to be an important part of human nutrition as they contain quercetin glycoside up to 250 mg/kg DW [41]. Investigated apples from Pakistan had 460 mg/kg DW of flavonols, with most prominent being myricetin [42]. Wild apple (*Malus prunifolia*) has also been investigated [43] and found to be a more potent antioxidant than (*Malus domestica*) apple. In *Malus prunifolia*, the rutin, hyperoside, and quercetin contents were found to be in the range 1.87–46.67 mg/L, 6.40–160.0 mg/L, and 3.33–83.33 mg/L, respectively [44]. Quercetin glycosides and dihydrochalcones were the predominant phenolic compounds in analyzed apple pomace (by-product of apple processing industries) from Spain. The most abundant compounds from the dihydrochalcone group were phloridzin (phloretin 2′-*O*-glucoside) and phloretin-2′-xyloglucoside, representing around 20% and 10%, respectively, of total phenolic compounds [45]. Phloridzin is the major dihydrochalcone in *Malus* species. In investigated apple peel, the concentration of phloridzin ranged from 24 to 825 µg/g, representing 3% to 93% of the total measured phenolics [46].

Raspberries are among the berry fruits that are the most represented in European countries. Ellagitannins and ellagic acid in raspberries are reported to have anticancer effects [47]. For 19 investigated raspberry cultivars, the concentration of free and hydrolyzed ellagic acid is reported to be in the range 120–320 mg/100 g FW [48]. In strawberries from Pakistan, high levels of flavonols were observed (3575 mg/kg DW), with a myricetin content of 3383 mg/kg DW, and kaempferol concentration 193 mg/kg DW [42]. Anthocyanins were the most abundant phenolic compounds in strawberries grown in Norway and their amount ranged from 8 to 66 mg/100 g of fresh weight (FW), while flavan-3-ols were found to be up to 45 mg/100 g of FW [49]. Raspberry, Arctic bramble, and cloudberry (all members of the *Rubus* family) extracts along with strawberry extract were shown to be effective inhibitors of pancreatic lipase in vitro [50]. In European blueberry (*Vaccinium myrtillus* L.) the highest amount of total analyzed anthocyanins was ascribed to delphinidin and cyanidin glycosides, while the mean content of total anthocyanins was 3768 mg/kg FW [51]. Mulberry (*Morus alba*) fruits grown in Serbia had rutin contents in the range 2.65–7.92 mg/kg of the frozen sample and remarkable concentrations of protocatechuic, chlorogenic, and ferulic acids [52].

Aronia berries or black chokeberries are also very good sources of polyphenol compounds, like anthocyanins, hydroxycinnamic acids, and proanthocyanidins [53]. Black chokeberry proanthocyanidins showed significant antimicrobial activity against 10 investigated pathogens [54]. Proanthocyanidins of *Aronia melanocarpa* berries are reported to reach the colon due to poor absorption in the gastrointestinal tract. Their action there can be manifested, because numerous phagocytic cells normally live in the gastrointestinal tract. Hence, black chokeberry proanthocyanidins could directly express their antioxidant and antimicrobial effects in vivo [54].

Phenolic profiles of 13 examined grapevine varieties from Serbia revealed flavan-3-ols to be the prominent phenols in grape seeds, and among them gallocatechin gallate and catechin were the most abundant [55]. Flavonols, quercetin, and myricetin were the primary phenols found in grape skins. Among anthocyanins in the berry skin were found 20 derivatives of delphinidin, malvidin, peonidin, petunidin, and cyanidin [55]. Other research showed ellagic acid in amounts up to 770 mg/kg and rutin up 450 mg/kg of dry weight, documenting that grapevine leaves are great source of polyphenols [56].

Rutin and chlorogenic acid were predominant polyphenols found in the Serbian autochthonous cultivar of sour cherry, along with pinobanksin, hesperetin, and galangin [57]. Cyanidin derivatives were the most abundant among sour cherry anthocyanins [57]. Naringenin and apigenin were found to be characteristic for fruit wine made from “Oblačinska” sour cherry [58].

Although a large number of different types of polyphenols are ingested through food, their absorption is very low and only about 1/10th of ingested polyphenols are absorbed through the stomach and intestine [59]. However, polyphenols must be chemically altered by microbiota and enzymes from the intestinal epithelium to be absorbed [60]. The antioxidant properties of phenolic compounds are related to the radical scavenging of free radicals, metal-chelating reactions, and modulation of cellular signaling pathways and gene expression [61,62]. Polyphenols inhibit or stimulate various enzymes, such as Akt/protein kinase B (Akt/PKB), phosphoinositide 3-kinase (PI3-kinase), protein kinase C (PKC), tyrosine kinase, mitogen-activated protein kinase (MAP kinase), and many others [63]. In addition, polyphenols from food increase the levels of two potent antioxidants in humans, such as uric acid and the enzyme paraoxonase [64,65].

However, there is still a debate over the action of polyphenols from the food in the body: epidemiological studies proved many positive health effects of polyphenols ingestion; on the other hand, due to the little resorption and rapid transformation, the bioavailability of polyphenols is relatively low.

### 2.1. Resveratrol

The polyphenol resveratrol belongs to the stilbene group; it belongs to phytoalexins, substances that plants produce in response to mechanical injury, UV radiation, and microbial and fungal infections [66]. Resveratrol is found in about 70 plant species; the highest amounts are present in grapes (about 3.5 mg/kg) and peanuts (about 1.9 mg/kg). The skin of red grapes contains 50 to 100 mg/kg resveratrol (fresh weight) [67].

Epidemiological studies show that resveratrol reduces the risk of cardiovascular diseases (CVD). Consumption of resveratrol-rich grape supplements during one year improved the inflammatory status in patients at high risk for CVD (with diabetes or hypercholesterolemia); it is recommended that resveratrol can be used as the primary prevention of CVD [68]. Absorption of resveratrol was proven to be better from grape products than from tablets [69].

Resveratrol exhibits anti-inflammatory and antioxidant activity, with antitumor and immunomodulatory effects [70]. This polyphenol compound was found to suppress the growth of 30 different tumors, such as tumors of the lung, liver, breast, prostate, colon, and esophagus [71].

The inhibitory effect of resveratrol on a number of enzymes such as cyclooxygenase, hydroperoxidase, protein kinase C, Bcl-2 phosphorylation, AKT, focal adhesion kinase, NFκB, matrix metalloprotease-9, and cell cycle regulators has been demonstrated [72]. Unfortunately, resveratrol has low bioavailability, below 1%, due to intense metabolism in the gut and liver. Plasma resveratrol is undetectable 30 to 120 min after oral administration of 25 mg of resveratrol [73]. Beside low bioavailability there are other limiting factors that affect resveratrol utilization. Exposure of resveratrol to the oxygen, oxidative enzymes and light may cause isomerization from the trans to the cis analogue with reduced activity [74,75].

#### 2.1.1. In Vitro Experiments

Many experiments proved that resveratrol inhibited proliferation in PC cells and induced apoptosis, probably via an inhibitory effect on the Hedgehog (Hh) signaling pathway [76,77,78]. In addition, resveratrol inhibits self-renewal capacity of cancer stem cells (CSCs) and stimulates apoptosis, via activation of caspases and inhibition of expression of Bcl-2, an apoptosis-inhibiting protein [79].

It was proven that resveratrol changed the factors involved in epithelial-to-mesenchymal transition (EMT), via the PI-3K/Akt/NF-κB signaling pathway, and suppressed the proliferation, invasion, and migration of PC cells [80]. Probably, resveratrol plays a dual role in PC: it suppresses tumor growth via Bax stimulation, but also stimulates tumor growth via vascular endothelial growth factor B (VEGF-B) activation, with the first effect being dominant [81]. Metformin, which is a potent inhibitor of VEGF-B, potentiates the antitumor effects of resveratrol [82].

A very important characteristic of resveratrol is its capability to sensitize PC cells to the effects of chemotherapeutics, including gemcitabine, a standard treatment for advanced PC patients. In addition, it was reported that resveratrol is capable of overcoming the chemoresistance during gemcitabine treatment [83,84]. Likely, the mechanism of action is via modification of signaling pathways, such as NFkB and STAT-3 [85]. The nuclear factor kB (NF-κB) is a protein complex that is involved in the cellular response to various types of stress. NF-κB controls DNA transcription, cytokine production, and cell survival [86]. The signal transducer activator transcription (STAT-3) factor is abnormally active in most cancers, including PC [87]. STAT-3 is an activator of vascular endothelial growth factor (VEGF) and hypoxia inducible factor (HIF-1a). VEGF and HIF-1a stimulate neovascularization in many cancers, including PC [88]. Another mechanism by which resveratrol increases the sensitivity of PC cells to gemcitabine is via activation of AMP-activation protein kinase (AMPK) [89].

In combination with capsaicin, or doxorubicin, resveratrol has been found to potentiate the action of gemcitabine on PC [90,91].

The resveratrol precursor triacetylresveratrol (TRES) inhibits EMT and PC growth via the Shh (sonic hedgehog) signaling pathway. It is considered that TRES has a future as a cure for the prevention and treatment of PC, due to its better bioavailability than resveratrol [92,93].

#### 2.1.2. Animal Studies

Concentrations used in in vitro experiments are hard to reach in vivo, which casts doubt on the real efficiency of resveratrol. In humans and rodents, after oral ingestion, about 70%–80% of resveratrol is rapidly absorbed, via rapid diffusion in the gut. After absorption, resveratrol conjugates to glucuronides and sulfates, so the highest level of resveratrol in plasma occurs 30-60 min after oral administration [94]. Due to its hydrophobic nature, resveratrol has poor solubility. In order to find a more efficient compound, some polyhydroxilated analogs, like pinosylvin and pinostilbene, were investigated, but were shown to have very poor bioavailability [95,96,97]. Dimethylether derivative of resveratrol, pterostilbene, because of enhanced lipophilicity was shown to have better bioavailability and improved biological activity [98]. A comparative study in rats, with orally administered resveratrol and pterostilbene, showed 10-fold higher bioavailability of pterostilbene [99]. Pterostilbene is found in blueberries and grapes.

In laboratory mice and guinea pigs, oral gavage with resveratrol reduced tumor volume and the risk of induced PC [83,100,101]. Co-administration of piperine with resveratrol significantly enhanced the bioavailability of resveratrol in mice. Results of the investigation indicated that piperine inhibits glucuronidation, one of the main pathways in resveratrol metabolism, therefore slowing down its elimination [102]. Quercetin and alcohol were proven to not affect the absorption of resveratrol [103].

#### 2.1.3. Clinical Studies

In humans, the circulating resveratrol level is only 2% of the highest serum concentration of free resveratrol and its conjugates, after taking a single dose of 25 mg/70 kg body weight [73]. Clinical studies show that after taking 25 mg of resveratrol at least 70% is absorbed, reaching the maximum concentration in plasma of about 500 ng/mL. The plasma half-life of resveratrol is about nine hours, but only very small amounts of unchanged resveratrol, fewer than 5 ng/mL, were detected. The major factor affecting the low bioavailability of resveratrol is the very rapid sulfate conjugation in the gut and liver [104]. Casein nanoparticles loaded with resveratrol were used to improve oral bioavailability of this polyphenol compound up to 10 times compared to oral solution [105]. Emulsions and nanoemulsions have positive effects on the stability of resveratrol, and therefore their bioavailability. Resveratrol encapsulated in grape seed oil is more stable to UV radiation [106].

After taking 2.5 g resveratrol for one month, a significant decrease of insulin-like growth factor-1 (IGF-1) and insulin-like growth factor binding protein 3 (IGFBP-3) in human plasma was recorded. The decrease of IGF-1 and IGFBP-3 possibly reflects the chemopreventive and anticancer activity of resveratrol. Doses of resveratrol <1.0 g did not lead to side effects in gastrointestinal tract, unlike the doses ≥2.5 g [107]. Clinical studies in humans showed pterostilbene to be nontoxic to normal cells and well tolerated [108].

### 2.2. Quercetin

Flavonoids are a group of polyphenolic compounds, distinguished by a flavan structure with 15 C atoms. It was already mentioned that various polyphenols are characteristic to certain foods (resveratrol in grapes, flavanones in citrus fruit, ellagic acid in berries, phloridzin in apples); while some polyphenols, like quercetin, are represented in all types of food (vegetables, fruits, cereals, tea, and wine). Quercetin is an important aglycone flavonoid prominent in fruits and vegetables [41,42,109] produced by plants because of UV radiation [110]. Antioxidative and radical scavenging activity of quercetin is well known [111]. Quercetin acts by inhibiting free radicals, therefore increasing production of non-enzymatic antioxidants and works synergistically with antioxidant enzymes under in vivo conditions [111]. Quercetin, usually found in the glycoside form, can be transformed through the activity of b-glucosidases to the aglycone form [112]. Quercetin from onions is mostly attached to glucose, while in apples and tea plants are in conjugation with rutinose [113,114]. The sugar component in the quercetin glycoside affects its bioavailability [115]. Certain studies have proven that quercetin from onion is more bioavailable than from apples [115]. Water solubility and oral absorption of quercetin is low, but because of its lipophilic nature, bioavailability could be enhanced by a fatty food diet [116]. About a quarter of the aglycone form of quercetin could pass through the gastrointestinal tract while glycoside forms of this flavonoid with hydrophilic characteristics are absorbed only in the small intestine [117,118].

#### In Vitro and In Vivo Experiments

The effect of quercetin was investigated on pancreatic cancer stem cells using in vitro and in vivo models [119]. Substrate assays, fluorescence-activated cell sorting (FACS), and Western blot analysis confirmed that activity of ALDH1 decreased and apoptosis resistance returned upon activity of quercetin. In vivo experiments showed reduced proliferation, expression of cancer stem cell-markers, angiogenesis, and induction of apoptosis after quercetin administration, along with cancer stem cell-enriched xenografts inhibition of growth. Significantly, quercetin in combination with sulforaphane had a synergistic effect, while no toxicity was observed [119]. Possible toxic effects of quercetin are pro-oxidant activity, mitochondrial toxicity, and inhibition of enzymes involved in hormone metabolism [120,121]. Quercetin dose dependent toxicity investigated during a two year feeding experiment with rats showed a severe nephrotoxicity effect, and caused primarily benign tumors of the renal tubular epithelium (neoplasia) only in male rats F344/N [122,123]. Rats in these experiments were exposed to doses about 2000 mg quercetin/kg body weight/day, which could correspond to doses of 140 g per 70 kg person [124], which is far less than the amount that can be ingested by consuming fruits and vegetables. Formulation of quercetin in food-grade lecithin improved bioavailability in healthy human volunteers [125]. For quercetin and its metabolites, reported half-life values were between 11 and 28 h [126].

Quercetin effectively inhibited pancreatic cancer cell growth in vitro and in vivo [127]. Increasing apoptosis, quercetin inhibited the growth of two pancreatic cancer cell lines: MIA PaCa-2 and BxPC-3. Furthermore, by using the orthotopic xenograft model, authors provided evidence that orally administrated quercetin reached biologically active tissue levels. The results so obtained showed the potential positive effect of quercetin in patients with pancreatic cancer [127].

### 2.3. Flavanols

#### 2.3.1. Green Tea Flavanols

Flavanols are polyphenols that belong to the flavonoid group. The major flavanols in green tea are catechins, like epigallocatechin-3-gallate (EGCG), epigallocatechin (EGC), epicatechin-3-gallate (ECG), and epicatechin (EC). The most prevalent catechin is EGCG (60%), followed by EGC (20%), ECG (14%), and EC (6%) [128]. Green tea also contains proanthocyanidins, theaflavins, and tearubigins [129]. Catechins are monomers, the building blocks of polymers (proanthocyanidins) and higher-order polymers (anthocyanidins).

The positive effects of catechins on human health may be the result of the chelation of iron (Fe) and copper (Cu) ions. Drinking of tea is known to reduce the absorption of iron from food. Fe-chelating complexes between iron and polyphenols have been demonstrated in the gut during digestion [130]. EGCG has poor intestinal stability and therefore reduced absorption which further affects its bioavailability. Nanoparticles, nanoemulsions, and nanoliposomes have been applied as a delivery system of EGCG to improve the stability and biological activity of oral administrated tea-polyphenol related products [131].

##### In Vitro and In Vivo Studies

Green tea has been known to protect against cancer by promoting cell cycle arrest and apoptosis [132]. The most active component of green tea, EGCG, inhibits NO-synthase gene expression, as well as NO-synthase enzyme activity [133]. Tea polyphenols increase the expression of cyclin-dependent kinase (CDK) inhibitors, which prevent cell proliferation and act as suppressors of tumor growth [134]. There is evidence that green tea polyphenol (GTPs) toxicity at high doses is due to their pro-oxidative properties [135]. Hepatotoxicity of GTPs at high doses has been evidenced in experiments with animals and also in epidemiological surveys. The authors also pointed out the hepato-protective activity of GTPs but only in low and medium doses [135]. An experiment conducted on hamsters showed that lipid hydroperoxide included in a long term high fat diet might induce carcinogenesis in the pancreas and liver, while the intake of green tea catechins might increase oxidative stress and therefore have a negative effect on the pancreas [136].

Numerous molecular targets of green tea extract (GTE) have been identified [137]. The same investigation identified 32 proteins involved in gene regulation and metabolism of cancer cells, whose levels were significantly altered by the action of GTE. Furthermore, the same authors documented that GTE inhibited Akt (protein kinase B) activation, decreased the levels of the mutant p53 protein, and induced apoptosis and suppressed tumor growth.

##### Human Studies

It was proven that polyphenols from tea can be absorbed from the intestinal tract, and exert various effects throughout the body [138]. Among catechins, ECG has the longest half-life, of about 5 h [139]. A dose of 1.0 g/m^2^ of GTE, three times a day, does not produce any side effects. This dose is equivalent to 7 to 8 Japanese cups, containing 120 mL of green tea [140].

However, to date, most epidemiological studies have not found clear confirmation that green tea drinking reduces PC risk [141,142].

#### 2.3.2. Grape Flavanols

##### In Vitro Experiments

Grape seed proanthocyanidins (GSPs) inhibited cell migration in human PC cell lines and decreased NF-κB inactivation. In addition, GSPs reversed EMT process and inhibited cell migration [143]. In addition, GSPs demonstrated the ability to modulate miRNA expression and to act similar to chemotherapeutic agents [144,145].

It was proven that GSPs inhibited cell proliferation and increased apoptosis in PC cell cultures. The main GSP actions were downregulation of the antiapoptotic protein Bcl-2 and mitochondrial membrane depolarization. In addition, GSPs decreased the formation of reactive oxygen species. Gallic acid possesses the highest antiproliferative and proapoptotic activity in the GSPs [146].

##### Clinical Studies

A study from Northern Italy, from 1991 to 2008, proved that proanthocyanidins intake was inversely related to PC risk. It was estimated that every-day eating of an additional portion of fruits rich in proanthocyanidins, such as apples, pears, grapes, and pulses, reduces the risk of PC by 25% [147].

##### Diabetes Mellitus, Pancreatic Cancer, and Flavanols

Recent research has linked diabetes mellitus (DM) to some hormone-dependent malignant diseases, such as pancreatic, colorectal, and breast cancer. It is thought that DM is linked to cancer by insulin resistance, hyperglycemia, and inflammation [148]. Most likely, hyperglycemia increases the invasive and migratory activities of PC cells [149].

Natural flavonoids with anti-diabetic effects can reduce the complications of DM, through the regulation of glucose and lipid metabolism and the activation of hepatic enzymes [150]. Regular and moderate use of wine, or grape juice, reduces the risk of type 2 diabetes (T2D) [151,152]. Wine polyphenols have a beneficial effect on DM and pancreatic cells through several mechanisms such as protection against glucose toxicity, inhibition of digestive enzymes, and anti-inflammatory effects [153,154,155].

### 2.4. Curcumin

**Figure 3:**
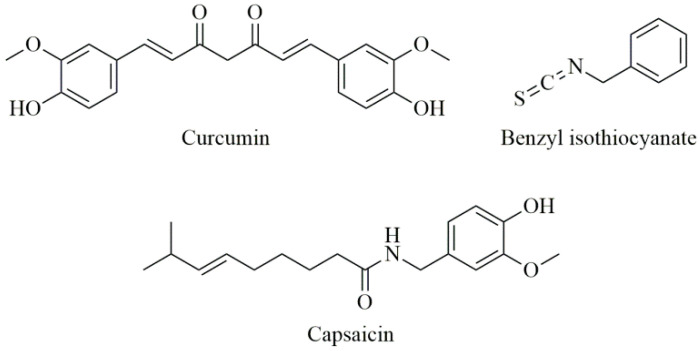
Chemical structures of curcumin, benzyl isothiocyanate, and capsaicin.

Curcumin (Figure 3) is a natural product isolated from the plant curcuma (*Curcuma longa*), which is commonly used in Indian cuisine. Curcumin has long been known for its antioxidant, anti-angiogenic, and anti-inflammatory effects and possible preventative effects on cancer [156].

Curcumin modulates a great number of signaling pathways, like NFkB, STAT-3, SP1, Notch-1, COX-II, ATM/Chk1, and WT1, and thus suppresses the growth of PC [157,158,159,160].

Curcumin is safe for humans even in very high doses of 12 g per day. However, due to poor absorption, rapid metabolism, and elimination, curcumin has limited bioavailability [161]. In patients with gemcitabine-resistant PC, gemcitabine and curcumin increased the median survival time [162]. Combination of curcumin with compounds like lecithin or piperine, and formulations with different hydrophilic or chitosan nanoparticles, improved cellular uptake and favorably affected pharmacokinetic parameters [163]. The synthetic analogue, difluorinated-curcumin (CDF), reaches 10 times greater concentrations in the pancreas than curcumin [157].

### 2.5. Other Substances with Potential Effects on Pancreatic Cancer

A specific diet rich in antioxidants and omega-3 fatty acids plays a significant role in patients with PC who are exposed to external radiation. These substances are thought to be able to reduce the toxic effects of therapy, such as nausea and diarrhea. Omega-3 polyunsaturated fatty acids (n-3 PUFAs) from fish oil, such as docosahexaenoic acid (DHA) and eicosapentaenoic acid (EPA), are useful in reducing anorexia. Probiotics with *Lactobacillus* and *Bifidobacterium* are helpful in patients with neutrophilia [164].

Drinking coffee has a positive effect on recovery of the pancreas after damage of pancreatic beta cells. Most of these effects are the result of the synergistic action of caffeine and polyphenols. In addition, drinking coffee every day could decrease the risk of metabolic syndrome [165].

Cruciferous vegetables show chemopreventive activity against PC and other various human malignant tumors [166,167]. Cabbages have large amounts of glucosinolates, which are converted to isothiocyanates (ITCs), which are released when plant cells are cut, or chewed [168]. The intestinal flora plays the largest role in the release of ITC from glucosinolates. Benzyl isothiocyanate (BITC) is a substance found in cabbage, cauliflower, mustard, and horseradish (Figure 3). BITC can also inhibit signaling pathways such as AKT, STAT-3, HDAC, NFkB, etc., and thus inhibit PC growth [169,170]. As BITC decreases STAT-3 signaling, it is also hypothesized that it may inhibit neoangiogenesis in PC. BITC has been shown to reduce tumor growth in experimental mice [171].

Seaweed is a promising source of proteins, vitamins, and minerals. In addition, seaweed has a high concentration of catechins, EC, EGC, and gallic acids [172]. Brown algae polyphenols have been shown to have antitumor potential for PC, as well for therapy-resistant PC [173,174]. It is believed that polyphenols from seaweed may inhibit PC relapse through stem-cell signaling in residual cells [175]. Antitumor effects on PC cells were also found in aronia extract and the *Salvia chinensis* extract [176,177].

Capsaicin (Figure 3) is a derivative of homovanilic acid, present in chili peppers at a concentration of 0.1% to 1%. The studies demonstrate that capsaicin may inhibit tumor growth in vitro and in vivo [178], although it is still debated whether capsaicin is completely safe [179], and also affects the expression of several genes involved in cancer cell survival, growth arrest, angiogenesis, and metastasis [180].

Table 1 summarizes the effects of the aforementioned polyphenols and signaling pathways involved in the PC.

## 3. Synergism of Polyphenols and PC

Some polyphenols with well-known anti-inflammatory characteristics (e.g., resveratrol, epigallocatechin gallate, curcumin, and quercetin) may alleviate the occurrence of acute pancreatitis, and incorporation of polyphenols in nutritional formulas would benefit patients with inflammatory bowel disease (IBD) and acute pancreatitis [181].

**Figure 4:**
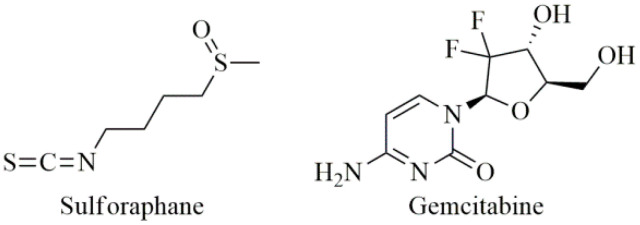
Chemical structures of sulforaphane and gemcitabine.

Early cancer metastases and aggressive growth can be caused by impaired regulation of stem cell self-renewal. Sulforaphane (SFN, Figure 4), mainly found in cruciferous vegetables, inhibits the self-renewal ability of PC stem cells (CSCs) and synergizes with quercetin, which is a predominant flavonoid in many fruits and vegetables especially in cruciferous vegetables [182]. The combination of quercetin and SFN had synergistic effects on the pancreatic CSCs’ self-renewal ability and that SFN alone or in combination with quercetin could eliminate the characteristics of cancer stem cells [183]. Furthermore, resveratrol and quercetin have been shown to inhibit PC cell line growth and are capable of reducing tumor cell invasion through the endothelial barrier, so they may be useful in the treatment of PC and the prevention of metastases [184].

The effects of gemcitabine (Figure 4) have been studied in the presence or absence of several polyphenols to evaluate whether they are able to potentiate the cytotoxicity of gemcitabine. Gemcitabine at 5 µg/mL in combination with 15µg/mL of some polyphenols (catechin, quercetin, bergamottin, rhamnetin) has been shown to be more effective than gemcitabine alone. Thus, the effect of gemcitabine chemotherapy can be significantly increased after combination with specific polyphenols, which may be promising agents for novel combination therapy [185].

## 4. Polyphenol Bioavailability

Bioavailability studies give insight what happens during ingestion, about the amounts of the compound capable to achieve systemic circulation, and what form of compound is present after the gastric and intestinal digestion. Bioavailability studies in vitro are derived from an in vitro bioaccessibility method, which takes into account the upper limit of a substance in food that could be released in the gastrointestinal tract and thus may become available for intestinal absorption. Chemical structure of polyphenols, along with their molecular weight, glycosylation, and esterification, affect their bioavailability [186]. Hydrophilic nature of phenolic aglycones is responsible for their diffusion through biological membranes and for their absorption. Polyphenols found in fruits and vegetables are mainly in the glycosidic form, which significantly affects their absorption in the intestines [187]. Therefore, the antioxidant activity or other biological activities in vitro do not necessarily mean that the compound will have biological activity, if a majority of the compound fails to reach a target tissue.

Bioaccessibility and cell uptake of phytochemicals of fresh citrus fruits has been investigated [188]. An experiment was done on human intestinal HepG2 cells, and during uptake, acids from the hydroxybenzoic group, along with hesperidin, narirutin, naringenin, and neohesperidin, were found in cells, while hydroxycinnamic acids and hesperitin were not detected in cells. Definitive conclusions on the bioavailability and bioactivity of a single phenolic compound are difficult to obtain, because of the possible antagonistic and synergistic effects of different citrus pulp components.

New in vitro dialyzability approaches applied in order to investigate total phenolics bioavailability in intestinal absorption process have been published [189]. Analyzed nuts and seeds with high phenolic contents, like walnuts and goji berries, showed low total phenolics dialyzability. In case of cashews, pistachios, and Brazil nuts with low/moderate total phenolic contents, high bioavailable ratios (80%–90%) have been found. Analysis of these results gave a correlation between TP bioavailability and Cu concentration in investigated nuts/seeds [189]. The most abundant phenolic compounds in our diet are not necessarily those that have the best bioavailability profile.

## 5. Conclusions

Pancreatic ductal adenocarcinoma continues to have a poor prognosis. Identifying ways to improve survival is a critical necessity. We are making progress as we learn more about the genomic alterations in pancreatic cancer. Modern drug design based on molecular profiling has promising results. However, alternative approaches, especially in the prevention strategies, come from research on the role of plant polyphenols in pancreatic cancer.

A large number of polyphenols have been shown to have potent antitumor, anti-inflammatory, antioxidant, and proapoptotic effects on cell cultures of various human cancers. However, the concentrations used in in vitro experiments exceed, sometimes thousands of times, the concentrations that can be used in humans. In addition, polyphenols are poorly absorbed in the human gastro- intestinal tract and are rapidly metabolized and excreted.

Therefore, future research could focus on a form of drug that would allow for better absorption and bioavailability of polyphenols. The application of nanoparticles is one approach to improve the bioavailability of polyphenols. Optimism is apparent in the first works studying nanoparticles, such as Ca-alginate, gold nanoparticles (AuNP), and zinc nanoparticles (ZnONP), which have shown cytotoxicity in pancreatic cancer [190,191,192].

## Figures and Tables

**Table 1:** Positive health activity of selected polyphenols and target/signaling pathways involved in PC.

Polyphenols	Activity and Signaling Pathways	Ref.
**Resveratrol and triacetylresveratrol**	Anti-inflammatory, antioxidant activity, antitumor and immunomodulatory; Cyclooxygenase, hydroperoxidase, protein kinase C, Bcl-2 phosphorylation, AKT, focal adhesion kinase, NFκB, matrix metalloprotease-9, cell cycle regulators, Hedgehog (Hh) and PI-3K/Akt/NF-κB signaling pathways, Bax stimulation.	[71,72]
**Quercetin**	Antioxidative and radical scavenging activity; Expression of cancer stem cell-markers, angiogenesis, and induction of apoptosis.	[119,126,127]
**Green tea flavanols**	Chelation of iron (Fe) and copper (Cu) ions; Cell cycle arrest, apoptosis, inhibition of NO-synthase gene expression and NO-synthase enzyme activity, Akt inhibition, decrease of mutant p53 protein.	[130,132,133,134,137]
**Grape flavanols**	Reversed EMT process, cell migration inhibition; miRNA expression modulation, decreased NF-κB inactivation, cell proliferation inhibition, apoptosis.	[143,144,145,146]
**Curcumin**	Antioxidant, anti-angiogenic and anti-inflammatory effects; Modulation fo NFkB, STAT-3, SP1, Notch-1, COX-II, ATM/Chk1, WT1.	[157,158,159,160]
**Benzyl isothiocyanate**	Chemopreventive activity; Inhibition of AKT, STAT-3, HDAC, NFkB.	[169,170]
**Capsaicin**	Inhibit tumor growth by NF-kB inactivation, ROS generations, cell-cycle arrest and modulating EGFR/HER-2 pathways	[178,180]
